# Association of growth with neurodevelopment in extremely low gestational age infants: a population-based analysis

**DOI:** 10.1007/s00431-022-04567-9

**Published:** 2022-07-22

**Authors:** Roland Gerull, Eva Huber, Valentin Rousson, Olaf Ahrens, Celine J. Fischer Fumeaux, Mark Adams, Cristina Borradori Tolsa, Roland P. Neumann, Myriam Bickle-Graz, Giancarlo Natalucci, for the Swiss Neonatal Network & Follow-up Group

**Affiliations:** 1 Department of Neonatology, University Children’s Hospital Basel UKBB, University of Basel, 4056 Basel, Switzerland; 2 Division of Neonatology, University Children’s Hospital, University of Bern, Berne, Switzerland; 3 Division of Biostatistics, Center for Primary Care and Public Health (Unisanté), University of Lausanne, Lausanne, Switzerland; 4 Department Mother-Woman-Child, Clinic of Neonatology, Lausanne University Hospital and University of Lausanne (CHUV), Lausanne, Switzerland; 5 Department of Neonatology, Newborn Research, University of Zurich and University Hospital Zurich, Zurich, Switzerland; 6 Division of Development and Growth, Department of Woman, Child and Adolescent, University Hospital, Geneva, Switzerland; 7 Family Larsson-Rosenquist Foundation Centre for Neurodevelopment, Growth and Nutrition of the Newborn, Department of Neonatology, University of Zurich and University Hospital Zurich, Zurich, Switzerland

**Keywords:** Growth, Development, Preterm, ELGAN

## Abstract

To assess the association between postnatal growth and neurodevelopment at the age of 2 years in extremely low gestational age newborns (ELGAN, < 28 weeks’ gestation). Retrospective population-based cohort study including all live born ELGAN in 2006–2012 in Switzerland. Growth parameters (weight, length, head circumference, body mass index) were assessed at birth, at hospital discharge home, and 2-year follow-up (FU2). Unadjusted and adjusted regression models assessed associations between growth (birth to hospital discharge and birth to FU2) and neurodevelopment at FU2. A total of 1244 infants (mean GA 26.5 ± 1.0 weeks, birth weight 853 ± 189 g) survived to hospital discharge and were included in the analyses. FU2 was documented for 1049 (84.3%) infants. The mean (± SD) mental and a psychomotor development index at 2FU were 88.9 (± 18.0) and 86.9 (± 17.7), respectively. Moderate or severe neurodevelopmental impairment was documented in 23.2% of patients. Changes of *z*-scores between birth and discharge and between birth and FU2 for weight were − 1.06 (± 0.85) and − 0.140 (± 1.15), for length − 1.36 (± 1.34), and − 0.40 (± 1.33), for head circumference − 0.61 (± 1.04) and − 0.76 (± 1.32) as well as for BMI 0.22 (± 3.36) and − 0.006 (± 1.45). Unadjusted and adjusted analyses showed that none of the four growth parameters was significantly associated with any of the three outcome parameters of neurodevelopment. This was consistent for both time intervals.

*Conclusion*: In the present population-based cohort of ELGAN, neither growth between birth and hospital discharge nor between birth and FU2 were significantly associated with neurodevelopment at age of 2 years.

**Table:** 

**What is Known:** *• Studies assessing the association between growth and neurodevelopment in extremely low gestational age newborns (28 weeks’ gestation) show conflicting results.*	
**What is New:** *• Neither growth between birth and hospital discharge nor between birth and corrected age of 2 years were significantly associated with neurodevelopment at age of 2 years.* *• The role of postnatal growth as a predictor of neurodevelopmental outcome during infancy might be smaller than previously assumed.*

**Supplementary Information:**

The online version contains supplementary material available at 10.1007/s00431-022-04567-9.

## Introduction

Around 10% of newborns worldwide are born preterm (less than 37 weeks’ gestation) [1] and around 0.5% are extremely low gestational age neonates (ELGAN, < 28 0/7 weeks’ gestation) [2]. In these patients significant improvements have led to decreased mortality rates over the last decades, but the risk for impaired cognitive and motor development remains substantial [3, 4]. Impairment affects not only infancy, but also has a relevant negative impact on school performance, academic achievement, and mental health later in adulthood [5–7].

While several risk factors for poor neurodevelopmental outcomes have been described such as low gestational age, brain lesions, bronchopulmonary dysplasia, proven sepsis [8], reports on the association of postnatal growth with neurodevelopment show conflicting results [9–13]. Malnutrition is negatively associated with brain development from early infancy into adulthood [14] and an association between decreased head growth and impaired neurodevelopment has been shown [15]. However, the impact of postnatal growth on neurodevelopment is still a matter of debate. While increasing caloric intake appears to improve growth, it might lead to increased body fat without improved lean body mass and to long-term adverse health outcomes such as increased risk for metabolic syndrome as adults [16].

We conducted a retrospective, population-based analysis of a large cohort of ELGAN to assess whether somatic growth during NICU stay and during the first 2 years of life is associated with neurodevelopmental outcome at the age of 2 years.

## Materials and methods

This is a retrospective population-based cohort study including live-born ELGAN who were born between 2006 and 2012 and were registered in the Swiss national registry of very preterm infants of the Swiss Society of Neonatology (SwissNeoNet, SNN). Infants with major congenital anomalies potentially affecting life expectancy or neurodevelopment (genetic anomaly or syndrome, or malformation of a major organ system), infants with primary non-intervention or palliative care at birth, and infants who died before hospital discharge were excluded.

The SNN prospectively collects perinatal, neonatal, and neurodevelopmental follow-up data of live born infants with a gestational age between 22 0/7 weeks and < 32 0/7 weeks or a birth weight of < 1501 g. All nine Swiss perinatal centers, five step-down units, and 16 neuro-/developmental pediatric units participate in the network. Since 2000, routine neurodevelopmental follow-up of preterm infants < 32 weeks of gestational age at a corrected age of 18 to 24 months (2-year follow-up, FU2) has been recommended.

Data collection and evaluation for this study were approved by the Swiss Federal Commission for Privacy Protection in Medical Research and the Swiss ethical review boards (KEK-ZH-Nr. 2014–0551 and KEK-ZH-Nr. 2014–0552). According to ethical review boards, no written parental informed consent was required for this study. However, the patients’ representatives were informed about the use of data for research. The study was performed in accordance with the Declaration of Helsinki and applicable local regulatory requirements.

### Growth measures

The anthropometric measures weight, length, head circumference, and body mass index (BMI, body mass divided by the square of the body height) were measured at birth, discharge home (not inter-hospital transfer) and at FU2. Neonatal and FU2 measures were recorded as absolute values and *z*-score according to Voigt [17] and Braegger [18], respectively.

### Sociodemographic, perinatal, and neonatal variables

Socio-economic status (SES) was classified according to Largo et al. [19]. Further demographic, perinatal, and neonatal baseline characteristics such as gestational age and major neonatal morbidities as listed in the results section and the supplemental material have been defined as previously described [20]. Major brain lesion was defined as either IVH grade III or IV, or cystic PVL. No data on feeding strategies or nutritional intake were available.

### Neurodevelopmental assessment at corrected age of 2 years

The FU2 neurodevelopmental assessments were performed by experienced pediatric neurologists or developmental pediatricians in one of the 16 centers of the Swiss Neonatal Follow-up Group. Tests were performed by means of the Bayley Scales of Infant Development, Second Edition (BSID-II) [21], the Bayley Scales of Infant and Toddler Development, Third Edition (Bayley-III) [22]. A subset of patients were tested using the Griffiths Mental Development Scales-Revised (GMDS)[23], or neurologic examination only. Vision and hearing were assessed by direct examination or caregiver report. Infants who were so severely impaired that structured testing was impossible were assigned a development score (whatever type) below − 3 standard deviations (SD) from the mean. Cerebral palsy (CP) was defined and graded according to Rosenbaum et al. [24] and to Palisano et al. [25].

### Outcomes

Moderate to severe neurodevelopmental impairment (NDI) at FU2 was defined as one of the following: mental or motor development index below 70 (− 2SD) in the BSID-II; cognitive or motor composite score below 85 in the Bayley-III, according to previous literature [26, 27]; a global score of the GMDS below 70 (− 2SD); cerebral palsy with GMFCS above 1; the absence of useful hearing even with aids (i.e., > 90 dB hearing level); blindness or only perception of light.

### Statistical methods

Primary and secondary analyses assessed the association between growth from birth to FU2 (delta2) as well as from birth to hospital discharge (delta1), respectively, and neurodevelopment at FU2. The association between the absolute anthropometric measures at birth, at hospital discharge, and FU2 were calculated post hoc.

We considered four (continuous) growth parameters (body weight, length, head circumference, and BMI) over the two periods delta1 and delta2, resulting in eight growth variables. We analyzed one binary outcome (NDI) and two additional outcomes derived from BSID-II (mental and psychomotor development index, MDI, PDI). This resulted in 24 analyzed associations. While analyses on NDI were based on all included infants, analyses of MDI and PDI included only patients assessed with BSID-II.

To reduce their spurious influence, a few outliers were set back to an extreme quantile of the distribution (winsorization). Associations with growth parameters were summarized via an odds-ratio, estimated from a logistic regression model (for the outcome NDI), or via a beta coefficient (slope) of a linear regression model (for the outcomes MDI and PDI). Odds ratios are to be interpreted as a ratio of odds of being diagnosed NDI, and beta coefficients as an average increase of the outcome, corresponding to a one-unit increase of the growth parameter.

To consider a possible clustering effect, we introduced a random «center effect» in all models, yielding (generalized) linear mixed models. To study whether the investigated associations could be the result of some confounding factors, we also performed adjusted analyses, including nine known risk factors for neurodevelopmental impairment in our models as available from the database (gestational age, sex, multiple birth, bronchopulmonary dysplasia, sepsis, necrotizing enterocolitis, retinopathy of prematurity, socio-economic status, and major brain lesion), yielding adjusted odds ratios and adjusted beta coefficients.

To deal with multiple testing issues, we pre-specified to apply a Bonferroni correction, resulting in *p*-values below 0.05/24 = 0.002 considered significant. All our models have been calculated using the *glmer routine* from the *lme4 package* available in the free statistical software R (R: A language and environment for statistical computing. R Foundation for Statistical Computing, Vienna, Austria; version 3.3.3).

## Results

A total of 2007 infants < 28 weeks’ gestation were born alive in Switzerland between 2006 and 2012 and therefore eligible for this study. Comparison with the Swiss Federal Statistical Office revealed 91% population coverage between 2007 and 2012 (reference data for 2006 were not available, as GA was not included in the national registry before 2007).

**Fig. 1:**
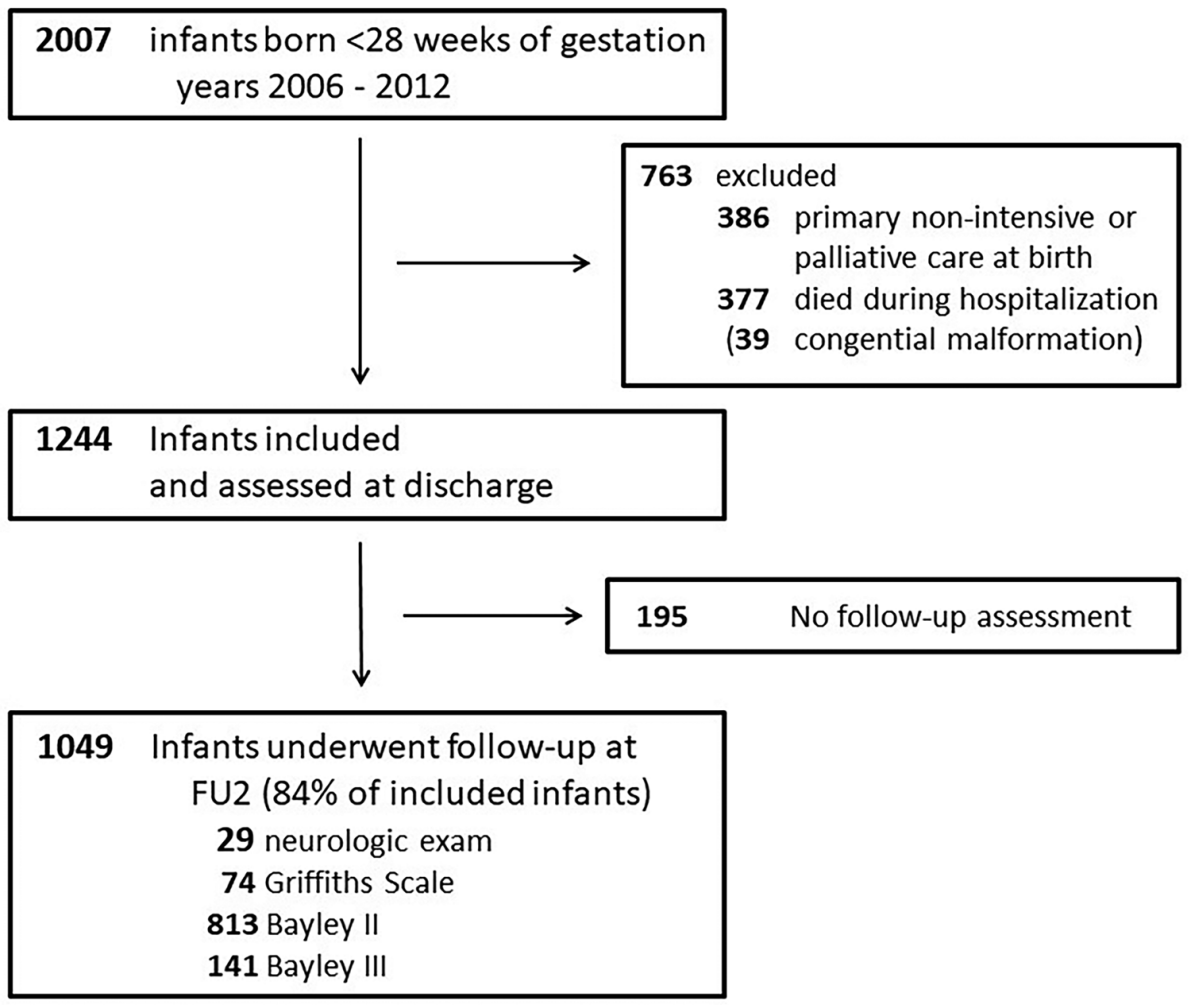
Flow chart of patient inclusion

After applying predefined exclusion criteria, 763 patients were excluded. The remaining 1244 patients had documented anthropometric measures at discharge and were included in analyses. Of these, 1049 (84.3%) had a documented neurodevelopmental assessment at FU2 (BSID-II, *n* = 813; Bayley-III, *n* = 141, GSID, *n* = 74; only neurological exam, *n* = 21) and were therefore eligible for further analyses (Fig. 1).

11.3% and 11.9% of included infants had a weight below the 10^th^ percentile at birth and FU2, respectively.

In comparison with infants without FU2 assessment, included infants had lower mean (SD) GA (26.4 weeks (± 1.0) vs 26.6 weeks (± 1.0), *p* = 0.014), lower mean birth weight (846 g (± 188) vs. 888 g (± 185), *p* = 0.006), and longer duration of supplemental oxygen (43.5 days (± 34.0) vs. 37.3 days (± 33.0), *p* = 0.015). Other perinatal and neonatal baseline characteristics (antenatal corticosteroids multiple birth, sex, rate of bronchopulmonary dysplasia, retinopathy of prematurity, major brain lesions, sepsis, patent ductus arteriosus, 1-min and 5-min Apgar, duration of hospitalization and parental SES) did not differ between groups with and without FU2 (Supplemental Table 1).

**Table 1:** Growth parameters of the study infants at birth, at hospital discharge and at FU2

	**Birth** **(*n* = 1244)**	**Hospital** **discharge** **(*n* = 1244)**	**FU2** **(*n* = 1049)**	**delta1:** **from birth** **to hospital** **discharge**	**delta2:** **from birth** **to FU2**
**Weight (g)**	853 (± 189)	2549 (± 837)	11320 (± 1706)	1699 (± 851)	10470 (± 1646)
**Weight** **z-score**	− 0.12 (± 0.88)	− 1.18 (± 1.01)	− 0.28 (± 1.16)	− 1.06 (± 0.85)	− 0.140 (± 1.15)
**Length (cm)**	34.3 (± 2.7)	47.1 (± 3.1)	84.5 (± 4.6)	13.0 (± 4.0)	50.4 (± 4.6)
**Length** **z-score**	0.12 (± 0.88)	− 1.29 (± 1.42)	− 0.35 (± 1.24)	− 1.36 (± 1.34)	− 0.40 (± 1.33)
**HC (cm)**	24.1 (± 1.6)	33.1 (± 2.8)	47.9 (± 1.8)	9.1 (± 3.1)	23.9 (± 1.8)
**HC** **z-score**	0.07 (± 0.99)	− 0.49 (± 1.11)	− 0.68 (± 1.38)	− 0.56 (± 1.04)	− 0.76 (± 1.32)
**BMI** **(kg/m**^**2**^**)**	7.2 (± 1.0)	12.8 (± 1.7)	15.8 (± 1.5)	5.6 (± 1.9)	8.6 (± 1.6)
**BMI** **z-score**	− 0.37 (± 2.02)	− 0.06 (± 3.21)	− 0.35 (± 1.13)	0.22 (± 3.36)	− 0.00 (± 1.45)

*FU2* 2-year follow-up examination at 18–24 months corrected age, *delta1* difference of growth parameters between birth and hospital discharge, *delta2* difference between birth and FU2, *HC* head circumference, *BMI* body mass index

Among all infants tested at FU2, 243 (23.2%) had moderate to severe NDI. The infants tested with the BSID-II, showed a mean (± SD) MDI and PDI of 88.9 (± 18.0) and 86.9 (± 17.7), respectively. Among the minority of infants tested with other tests, the mean scores of the cognitive and motor composite of the Bayley-III were 99.0 (± 38.0) and 96.4 (± 36.7), respectively. The global score of the GSID was 83.6 (± 24.8).

The mean body weight of participants was 853 g (± 189) at birth, 2549 g (± 837) at hospital discharge, and 10470 g (± 1646) at FU2. Corresponding weight *z*-scores were − 0.12 (± 0.88), − 1.18 (± 1.01), and − 0.28 (± 1.16), respectively. The body length *z*-scores for birth, discharge, and FU2 were 0.12 (± 0.88), − 1.29 (± 1.42), and − 0.35 (± 1.24), respectively. Head circumference *z*-score decreased during the observation period with lowest values at FU2. Detailed anthropometric values of the study participants are shown in Table 1.

### Primary analyses

Unadjusted and adjusted regression analyses showed no statistical evidence of associations between growth from birth to FU2 and neurodevelopment at FU2 according to the predefined level of significance (*p* < 0.002). None of the four growth parameters was significantly associated with any of the three outcome parameters of development (NDI, MDI, and PDI) (Tables 2 and 3).

**Table 2:** Unadjusted and adjusted association of growth between birth and hospital discharge (delta1) and between birth and FU2 (delta2) with neurodevelopmental impairment at 18- to 24-month corrected age

	**Unadjusted regression**		**Adjusted regression**	
	Odds ratio (95% CI)	*p*-value	Odds ratio (95% CI)	*p*-value
**delta1 weight** ***z-*score**	0.90 (0.74, 1.10)	0.2963	0.95 (0.77, 1.17)	0.6415
**delta1 length** ***z-*score**	0.99 (0.84, 1.18)	0.9252	1.00 (0.83, 1.21)	0.9779
**delta1 HC** ***z-*score**	1.02 (0.86, 1.21)	0.7890	1.01 (0.84, 1.21)	0.9265
**delta1 BMI** ***z-*score**	1.13 (0.98, 1.30)	0.0840	1.04 (0.89, 1.21)	0.6206
**delta2 weight** ***z-*score**	0.84 (0.74, 0.96)	0.0083	0.83 (0.72, 0.95)	0.0069
**delta2 length** ***z-*score**	0.96 (0.85, 1.07)	0.4471	0.94 (0.83, 1.06)	0.3129
**delta2 HC** ***z-*score**	0.93 (0.83, 1.04)	0.1913	0.95 (0.84, 1.07)	0.4137
**delta2 BMI** ***z-*score**	0.93 (0.84, 1.03)	0.1545	0.90 (0.81, 1.01)	0.0723

In adjusted analysis, odds ratios are adjusted for gestational age, sex, multiple births, bronchopulmonary dysplasia, sepsis, necrotizing enterocolitis, retinopathy of prematurity, socio-economic status, and major brain lesion

*delta1* difference between birth and hospital discharge, *delta2* difference between birth and FU2, *HC* head circumference, *BMI* body mass index

**Table 3:** Unadjusted and adjusted association between growth parameters at birth and at hospital discharge (delta1) and at birth and at FU2 (delta2) with mental development intex (MDI) and psychomotor development index (PDI) at 18- to 24-month corrected age

	**Unadjusted regression**		**Adjusted regression**	
	**MDI**			
	*β* (95% CI)	*p*-value	*β* (95% CI)	*p*-value
**delta1 weight** ***z-*score**	− 0.33 (− 2.06, 1.39)	0.7036	− 0.16 (− 1.83, 1.51)	0.8516
**delta1 length** ***z-*score**	− 0.32 (− 1.71, 1.07)	0.6484	− 0.01 (− 1.34, 1.31)	0.9829
**delta1 HC** ***z-*score**	− 0.51 (− 1.88, 0.87)	0.4693	− 0.15 (− 1.49, 1.18)	0.8216
**delta1 BMI** ***z-*score**	− 0.85 (− 1.95, 0.24)	0.1254	− 0.19 (− 1.30, 0.91)	0.7308
**delta2 weight** ***z-*score**	0.07 (− 1.06, 1.20)	0.8985	0.55 (− 0.56, 1.66)	0.3303
**delta2 length** ***z-*score**	− 0.38 (− 1.34, 0.58)	0.4326	0.11 (− 0.84, 1.06)	0.8179
**delta2 HC** ***z-*score**	0.80 (− 0.19, 1.80)	0.1134	0.82 (− 0.15, 1.80)	0.0960
**delta2 BMI** ***z-*score**	0.01 (− 0.91, 0.93)	0.9753	0.39 (− 0.50, 1.28)	0.3912
	**PDI**			
	*β* (95% CI)	*p*-value	*β* (95% CI)	*p*-value
**delta1 weight** ***z-*score**	− 0.77 (− 2.50, 0.97)	0.3864	− 0.95 (− 2.69, 0.80)	0.2868
**delta1 length** ***z-*score**	0.43 (− 1.00, 1.87)	0.5525	0.39 (− 1.08, 1.85)	0.6055
**delta1 HC** ***z-*score**	− 1.08 (− 2.50, 0.35)	0.1392	− 1.06 (− 2.49, 0.37)	0.1474
**delta1 BMI** ***z-*score**	− 1.00 (− 2.10, 0.09)	0.0729	− 0.18 (− 1.30, 0.94)	0.7525
**delta2 weight** ***z-*score**	1.33 (0.18, 2.47)	0.0236	1.58 (0.42, 2.74)	0.0078
**delta2 length** ***z-*score**	− 0.05 (− 1.03, 0.93)	0.9210	0.15 (− 0.84, 1.14)	0.7651
**delta2 HC** ***z-*score**	0.16 (− 0.84, 1.16)	0.7586	0.08 (− 0.92, 1.08)	0.8755
**delta2 BMI** ***z-*score**	1.17 (0.24, 2.11)	0.0142	1.43 (0.50, 2.36)	0.0027

In adjusted analysis, beta values are adjusted for gestational age, sex, multiple births, bronchopulmonary dysplasia, sepsis, necrotizing enterocolitis, retinopathy of prematurity, socio-economic status, and major brain lesion

*HC* head circumference, *BMI* body mass index

### Secondary analyses

Growth between birth and hospital discharge was not significantly associated with neurodevelopment at FU2. Similar to primary analyses, no significant associations between any of the four growth parameters and neurodevelopmental parameters were detected (Tables 2 and 3).

### Post hoc analysis

Analyses of the association of the four anthropomorphic measurements assessed at three timepoints with the three neurodevelopment outcomes were performed post hoc, resulting in 36 analyses. Of these, 9 showed significant associations in adjusted analyses. In more detail, significant associations were detected between weight and length at FU2 with moderate to severe NDI (*p* = 0.0004 and *p* = 0.0007) as well as length at birth and head circumference at FU2 with MDI (*p* = 0.0019 and *p* = 0.0019). Furthermore, length and head circumference at birth, as well as weight, length, and BMI at FU2, showed significant associations with PDI (*p* < 0.0001, *p* = 0.0019, *p* < 0.0001, and *p* = 0.0002, respectively).

Detailed information about anthropometric measurements and neurodevelopment at FU2 is provided in the supplemental material.

Separate analyses of SGA (*n* = 141) and non-SGA (*n* = 1103) patients were also carried out and showed mostly no significant associations between growth parameters and development. In particular, none of the eight growth parameters considered showed a significant association with NDI. However, for SGA patients, the weight *z-*score difference between birth and hospital discharge was significantly associated with MDI in unadjusted and adjusted analyses (*p* = 0.0004 and *p* = 0.0003). Furthermore, the difference of weight *z-*score and BMI *z-*score between birth and FU2 was significantly associated with PDI in unadjusted and adjusted analyses (weight *p* = 0.0001 and 0.0001; BMI *p* = 0.0004 and 0.0002). In all these analyses, increased growth was associated with better development.

Detailed information about these analyses are provided in the supplemental material.

## Discussion

Analyses of this population-based cohort of extremely preterm born infants did not show statistical evidence that either growth between birth and discharge, or growth between birth and the age of 2 years were associated with 2-year neurodevelopmental outcomes.

The association between postnatal growth and neurodevelopment in preterm infants has been studied repeatedly over the last decades [10, 28]. However, considerable heterogeneity exists in the analyzed patient collective, sample size, primary outcomes, assessment measures and adjustment for confounding variables. For example, included patients were categorized as small for gestational age versus adequate for gestational age and growth was categorized according to quartiles of the normative values of the patient collective [10]. In both cases, analyses are less precise than analyzing growth parameters as continuous variables as performed in the present study. Furthermore, several studies measured growth as weight gain in gram/kg/d, which is not as precise as analyses of *z-*scores. Other studies did not adjust for confounding variables, had small sample size or did not focus on extremely preterm infants as our study. Thus, only a small number of studies analyzed the association between postnatal growth and neurodevelopment in extremely preterm infants precisely in a large collective.

Overall, the majority of publications imply that an association between postnatal growth and neurodevelopment exists, but some important limitations are present in most of them. In particular, only few studies adjusted development for socio-economic status, which is an important predictor of neurodevelopment [10].

The French EPIPAGE study documented increased risk of cognitive impairment and inattention-hyperactivity at the age of 5 years in preterm infants < 32 weeks who were born small for gestational age. Furthermore, impaired postnatal growth of patients with appropriate for gestational age birthweight was associated with cerebral palsy and school difficulties [12]. In contrast to our study, patients of higher gestational age were included and growth was assessed only at the age of 6 months. Moreover, eight outcome parameters were presented and no correction for multiple testing was applied.

Similar to our study Belfort et al. assessed the relationship between growth and neurocognitive development in a large American collective of ELGAN [9]. While presenting multiple analyses, lower weight gain was not associated with higher or lower risk of low MDI, low PDI, cerebral palsy or microcephaly. Significant associations with neurodevelopment were only detected in subsets of infants (weight *z-*score < − 2 at the age of 12 months, not considering growth).

Based on the abovementioned literature, it is difficult to draw strong conclusions about the impact of postnatal growth on neurodevelopment in the extreme preterm population. In light of the present findings, it seems that postnatal growth is not a predictor of neurodevelopment as described previously.

While *z-*scores for all four growth parameters declined between birth and hospital discharge, length and body weight showed a catch-up growth at FU2. Only head circumference showed a *z-*score below − 0.4 at FU2. Since patients with catch-up growth might have a better outcome than patients without catch-up growth [12], the growth rate might be an explanation for our results.

Growth and development were also studied in a Brazilian collective of very low birthweight (birthweight < 1500 g) infants. Similar to our results, this study showed that growth was not a significant predictor for neurodevelopment [29].

Post hoc analyses of SGA infants of the present study revealed that three growth parameters were associated with neurodevelopment. These associations were only detected in SGA patients, but not in non-SGA patients and not in the entire patient group. Therefore, future studies might focus specifically on SGA patients.

It is important to state that this study did not include intrauterine growth as a risk factor, although extensive studies have documented that intrauterine growth restriction is associated with impaired development [30–33].

Over the last decades, a major aim in neonatology was to improve growth of preterm infants to enable optimal development. However, results of our study question if focusing on weight gain improves neurodevelopment of extremely preterm infants. Moreover, a large number of studies have documented a positive association between postnatal weight gain and adiposity, insulin resistance as well as increased blood pressure [28, 34, 35]. Therefore, potential positive effects of increased weight gain have to be balanced against existing risks. In fact, current nutritional strategies do not aim to only improve growth, but to optimize the quantity and quality of the intake, to increase breast milk consumption, while trying to reproduce body composition resembling that of term infants as much as possible.

Important questions about optimal growth remain unanswered. In particular, nutritional status in preterm infants is very complex and measuring growth in grams and centimeters describes growth only quantitatively, but not qualitatively. Assessment of lean body mass or supply of micronutrients might give additional information about optimal growth.

The following weaknesses limit the generalizability of the present findings. First, the retrospective design of the study implies a reporting bias. Since patients who were assessed at FU2 were slightly sicker (see supl. Table 1) than patients who were not assessed, a bias seems possible.

Second, the study includes patients born 2006–2012 with FU2 not later than 2014, which limits generalizability of our results. Despite advances in neonatal care, several studies show that neither mortality nor the rate of neurodevelopment has improved substantially over the last decades. In fact, a recent meta-analysis concluded that no definite trend of improved neurodevelopment at school age for neurosensory, cognitive, academic achievement, motor or executive function exists [36, 37]. However, some studies suggest that the spectrum of NDI shifted towards less severe CP and less severe sensory impairment. Nevertheless, we speculate that more recent data would provide results comparable to our study.

Third, multiple testing required a Bonferroni correction. While maintaining the overall type I error at 5%, this also had the consequence of increasing the type II error, such that we could have missed a few significant results. In particular, the adjusted association between delta2 weight *z-*score and NDI was potentially clinically impactful, reaching a *p*-value of 0.0069. This would have been significant if we had chosen to investigate less associations. Similar remarks hold for the adjusted association between delta2 weight, respectively delta 2 BMI with PDI. It will be interesting to see whether such potential associations can be confirmed in future studies.

Fourth, postnatal development was assessed with three different tools. While the composite outcome NDI included the results of all three developmental assessment methods, we included a secondary analysis considering only BSID-II data focusing on the largest subgroup of the cohort studied.

The strengths of the present study include the large size of the cohort, the utilization of standardized neurodevelopmental measures, and the prospective nature of the dataset regarding a geographically defined population.

## Conclusion

Results of the present study show that growth between birth and hospital discharge, as well as growth between birth and age of 2 years, was not associated with impaired neurodevelopment in this Swiss cohort of extremely preterm infants. The role of postnatal growth as a predictor of neurodevelopmental outcome during infancy might be smaller than previously assumed.

## Supplementary Information

Below is the link to the electronic supplementary material.

Supplementary file1 (DOCX 17 KB)

Supplementary file2 (DOCX 17 KB)

Supplementary file3 (DOCX 16 KB)

Supplementary file4 (DOCX 16 KB)

Supplementary file5 (DOCX 15 KB)

Supplementary file6 (DOCX 16 KB)

## Data Availability

All data are available upon request. Please address to the corresponding author Roland Gerull.

## Abbreviations

BMI: Body mass index (body mass divided by the square of the body height)
BSID: Bayley Scales of Infant Development
CP: Cerebral palsy
ELGAN: Extremely low gestational age newborns (less than 1000 g)
FU2: Follow up at the corrected age of 2 years
GMDS: Griffiths Mental Development Scales-Revised
IVH: Intraventricular hemorrhage
MDI: Mental development index
NDI: Neurodevelopmental impairment
NICU: Neonatal intensive care unit
PDI: Psychomotor development index
PVL: Periventricular leukomalacia
SD: Standard deviation
SES: Socio economic status
SNN: Swiss Neo Net
ELGAN: Extremely low gestational age newborns (birth weight less than 1000g)
