# Supplementary material for: Association of growth with neurodevelopment in extremely low gestational age infants: a population-based analysis
Source: Eur J Pediatr. 2022 Jul 22;181(10):3673–81. doi: 10.1007/s00431-022-04567-9 (PMC9508205; doi:10.1007/s00431-022-04567-9)
Supplement: Supplementary file 1 — Supplementary file1 (DOCX 17 KB) [file 431_2022_4567_MOESM1_ESM.docx]

**Supplemental table 1: Baseline characteristics of infants assessed at age 2 years and loss of follow up**

|  | Infants with FU2  (n=1049) | Infants loss of follow up (n=195) | p-value |
| --- | --- | --- | --- |
| Gestational age (weeks) | 26.4 (±1.0) | 26.6 (±1.0) | 0.014 |
| Birth weight (grams) | 846 (±188) | 888 (±185) | 0.006 |
| Male (%) | 53.4 | 52.5 | 0.816 |
| Multiples (%) | 26.7 | 25.8 | 0.861 |
| Caesarean section (%) | 80.1 | 75.8 | 0.147 |
|  | | | |
| Apgar 1 Minute | 5.0 (±2.5) | 4.8 (±2.3) | 0.390 |
| Apgar 5 Minute | 7.1 (±1.8) | 6.9 (±1.9) | 0.310 |
| Surfactant (%) | 72.1 | 66.7 | 0.124 |
| Supplemental O_2_ (days) | 43.5 (±34.0) | 37.3 (±33.0) | 0.015 |
| BPD (%) | 22.2 | 20.7 | 0.708 |
| Confirmed sepsis (%) | 24.3 | 18.7 | 0.099 |
| PDA (%) | 52.6 | 49.0 | 0.353 |
| NEC (%) | 2.4 | 3.0 | 0.618 |
| ROP (%) | 5.9 | 4.9 | 0.731 |
| Major brain lesion (%) | 10.1 | 10.1 | 1.000 |
| Length of stay (days) | 91.8 (±30.5) | 86.9 (±27.4) | 0.114 |
|  | | | |
| Socio economic score | 6.2 (±2.5) | 6.2 (±2.6) | 0.621 |

Results are shown as percentage or mean (±standard deviation)

FU2, follow-up assessment at age 2 years; BPD bronchopulmonary dysplasia; PDA, patent ductus arteriosus; NEC, necrotizing enterocolitis; ROP, retinopathy of prematurity requiring treatment; Major brain lesion, intraventricular hemorrhage with ventricular dilatation and/or periventricular hemorrhage.
