# Supplementary material for: Association of growth with neurodevelopment in extremely low gestational age infants: a population-based analysis
Source: Eur J Pediatr. 2022 Jul 22;181(10):3673–81. doi: 10.1007/s00431-022-04567-9 (PMC9508205; doi:10.1007/s00431-022-04567-9)
Supplement: Supplementary file 2 — Supplementary file2 (DOCX 17 KB) [file 431_2022_4567_MOESM2_ESM.docx]

**Supplemental table 2: Association between somatic growth parameters at birth, at hospital discharge and at 2-year follow-up and moderate to severe neurodevelopmental impairment at age 2 years.**

|  | Unadjusted Analysis | | Adjusted Analysis | |
| --- | --- | --- | --- | --- |
|  | OR (95% CI) | p-value | OR (95% CI) | p-value |
| Weight  z-score | 0.90 (0.76, 1.05) | 0.1866 | 0.89 (0.75, 1.07) | 0.2183 |
| Length at birth  z-score | 0.81 (0.70, 0.93) | 0.0027 | 0.81 (0.69, 0.94) | 0.0060 |
| Head circumference at birth  z-score | 0.81 (0.70, 0.94) | 0.0064 | 0.78 (0.66, 0.92) | 0.0028 |
| BMI at birth  z-score | 1.00 (0.88, 1.14) | 0.9876 | 1.02 (0.89, 1.17) | 0.7795 |
| Weight at discharge  z-score | 0.80 (0.68, 0.94) | 0.0073 | 0.81 (0.69, 0.96) | 0.0174 |
| Length at discharge  z-score | 0.88 (0.76, 1.03) | 0.1140 | 0.89 (0.75, 1.05) | 0.1679 |
| Head circumference at discharge  z-score | 0.83 (0.72, 0.97) | 0.0182 | 0.79 (0.67, 0.94) | 0.0067 |
| BMI at discharge  z-score | 1.19 (0.99, 1.42) | 0.0602 | 1.02 (0.84, 1.24) | 0.8491 |
| Weight at FU2  z-score | 0.80 (0.70, 0.90) | **0.0004** | 0.78 (0.68, 0.90) | **0.0004** |
| Length at FU2  z-score | 0.82 (0.73, 0.92) | **0.0011** | 0.80 (0.71, 0.91) | **0.0007** |
| Head circumference at FU2  z-score | 0.84 (0.75, 0.93) | **0.0012** | 0.84 (0.75, 0.94) | 0.0035 |
| BMI at FU2  z-score | 0.87 (0.76, 0.99) | 0.0371 | 0.85 (0.74, 0.99) | 0.0307 |

BMI, body mass index; FU2, 2-year follow-up assessment.

In adjusted analysis, beta values are adjusted for gestational age, sex, multiple births, bronchopulmonary dysplasia, sepsis, necrotizing enterocolitis, retinopathy of prematurity, socio-economic status and major brain lesion.
