# Supplementary material for: Association of growth with neurodevelopment in extremely low gestational age infants: a population-based analysis
Source: Eur J Pediatr. 2022 Jul 22;181(10):3673–81. doi: 10.1007/s00431-022-04567-9 (PMC9508205; doi:10.1007/s00431-022-04567-9)
Supplement: Supplementary file 3 — Supplementary file3 (DOCX 16 KB) [file 431_2022_4567_MOESM3_ESM.docx]

**Supplemental Table 3: Association between somatic growth parameters at birth, at hospital discharge, and at 2-year follow-up and mental development index (BSID-II) at age 2 years.**

|  | Unadjusted Analysis | | Adjusted Analysis | |
| --- | --- | --- | --- | --- |
|  | β (95% CI) | p-value | β (95% CI) | p-value |
| Weight  z-score | 1.71 (0.33, 3.10) | 0.0151 | 1.19 (-0.20, 2.58) | 0.0921 |
| Length at birth  z-score | 2.44 (1.26, 3.61) | **<0.0001** | 1.86 (0.69, 3.03) | **0.0019** |
| Head circumference at birth  z-score | 2.01 (0.71, 3.31) | 0.0024 | 1.43 (0.14, 2.72) | 0.0296 |
| BMI at birth  z-score | 0.44 (-0.67, 1.55) | 0.4400 | -0.03 (-1.11, 1.05) | 0.9580 |
| Weight at discharge  z-score | 1.44 (0.04, 2.83) | 0.0439 | 1.24 (-0.09, 2.58) | 0.0685 |
| Length at discharge  z-score | 1.26 (0.01, 2.50) | 0.1140 | 1.14 (-0.06, 2.33) | 0.0633 |
| Head circumference at discharge  z-score | 1.09 (-0.19, 2.38) | 0.0944 | 0.92 (-0.33, 2.16) | 0.1487 |
| BMI at discharge  z-score | -1.23 (-2.65, 0.20) | 0.0908 | -0.32 (-1.77, 1.13) | 0.6641 |
| Weight at FU2  z-score | 1.16 (0.07, 2.26) | 0.0374 | 1.24 (0.17, 2.31) | 0.0230 |
| Length at FU2  z-score | 1.21 (0.20, 2.22) | 0.0194 | 1.40 (0.41, 2.39) | 0.0054 |
| Head circumference at FU2  z-score | 1.71 (0.78, 2.63) | **0.0003** | 1.45 (0.53, 2.36) | **0.0019** |
| BMI at FU2  z-score | 0.58 (-0.56, 1.72) | 0.3189 | 0.56 (-0.56, 1.68) | 0.3267 |

BMI, body mass index; FU2, 2-year follow-up assessment.

In adjusted analysis, beta values are adjusted for gestational age, sex, multiple births, bronchopulmonary dysplasia, sepsis, necrotizing enterocolitis, retinopathy of prematurity, socio-economic status and major brain lesion
