# Supplementary material for: Association of growth with neurodevelopment in extremely low gestational age infants: a population-based analysis
Source: Eur J Pediatr. 2022 Jul 22;181(10):3673–81. doi: 10.1007/s00431-022-04567-9 (PMC9508205; doi:10.1007/s00431-022-04567-9)
Supplement: Supplementary file 4 — Supplementary file4 (DOCX 16 KB) [file 431_2022_4567_MOESM4_ESM.docx]

**Supplemental Table 4: Association between somatic growth parameters at birth, at hospital discharge, and at 2-year follow-up and psychomotor development index (BSID-II) at age 2 years.**

|  | Unadjusted Analysis | | Adjusted Analysis | |
| --- | --- | --- | --- | --- |
|  | β (95% CI) | p-value | β (95% CI) | p-value |
| Weight  z-score | 2.22 (0.81, 3.62) | 0.0020 | 1.93 (0.48, 3.38) | 0.0091 |
| Length at birth  z-score | 2.88 (1.68, 4.09) | **<0.0001** | 2.56 (1.34, 3.79) | **<0.0001** |
| Head circumference at birth  z-score | 2.09 (0.78, 3.40) | **0.0017** | 2.12 (0.78, 3.46) | **0.0019** |
| BMI at birth  z-score | 0.08 (-1.07, 1.22) | 0.8974 | -0.17 (-1.32, 0.98) | 0.7684 |
| Weight at discharge  z-score | 1.88 (0.47, 3.29) | 0.0088 | 1.44 (0.05, 2.84) | 0.0426 |
| Length at discharge  z-score | 1.98 (0.71, 3.25) | 0.0022 | 1.82 (0.55, 3.09) | 0.0051 |
| Head circumference at discharge  z-score | 1.12 (-0.21, 2.45) | 0.0978 | 1.11 (-0.22, 2.44) | 0.1013 |
| BMI at discharge  z-score | -1.74 (-3.17, -0.30) | 0.0178 | -0.60 (-2.09, 0.88) | 0.4258 |
| Weight at FU2  z-score | 2.54 (1.46, 3.63) | **<0.0001** | 2.54 (1.45, 3.63) | **<0.0001** |
| Length at FU2  z-score | 1.97 (0.96, 2.97) | **0.0001** | 1.92 (0.91, 2.94) | **0.0002** |
| Head circumference at FU2  z-score | 1.09 (0.17, 2.01) | 0.0201 | 1.04 (0.11, 1.97) | 0.0286 |
| BMI at FU2  z-score | 1.85 (0.71, 2.99) | **0.0015** | 2.03 (0.87, 3.18) | **0.0006** |

BMI, body mass index; FU2, 2-year follow-up assessment.

In adjusted analysis, beta values are adjusted for gestational age, sex, multiple births, bronchopulmonary dysplasia, sepsis, necrotizing enterocolitis, retinopathy of prematurity, socio-economic status and major brain lesion
