# Supplementary material for: Association of growth with neurodevelopment in extremely low gestational age infants: a population-based analysis
Source: Eur J Pediatr. 2022 Jul 22;181(10):3673–81. doi: 10.1007/s00431-022-04567-9 (PMC9508205; doi:10.1007/s00431-022-04567-9)
Supplement: Supplementary file 5 — Supplementary file5 (DOCX 15 KB) [file 431_2022_4567_MOESM5_ESM.docx]

**Table 5: Analyses of small for gestational age (SGA) patients (n=141). Association between somatic growth parameters at birth, at hospital discharge, and at 2-year follow-up and moderate to severe neurodevelopmental impairment at the age of 2 years**

|  | unadjusted regression | | adjusted regression | |
| --- | --- | --- | --- | --- |
|  | Odds ratio  (95% CI) | p-value | Odds ratio  (95% CI) | p-value |
| delta1 weight  z-score | 0.59  (0.35, 1.00) | 0.0486 | 0.53  (0.30, 0.92) | 0.0255 |
| delta1 length  z-score | 0.89  (0.55, 1.44) | 0.6342 | 0.92  (0.54, 1.56) | 0.7546 |
| delta1 HC  z-score | 0.86  (0.55, 1.34) | 0.4995 | 0.80  (0.50, 1.28) | 0.3609 |
| delta1 BMI  z-score | 1.19  (0.80, 1.77) | 0.3886 | 1.05  (0.68, 1.63) | 0.8186 |
|  | | | | |
| delta2 weight  z-score | 0.74  (0.52, 1.05) | 0.0934 | 0.69  (0.48, 1.00) | 0.0506 |
| delta2 length  z-score | 0.90  (0.66, 1.23) | 0.5046 | 0.69  (0.64, 1.25) | 0.5303 |
| delta2 HC  z-score | 0.80  (0.60, 1.07) | 0.1341 | 0.85  (0.62, 1.17) | 0.3157 |
| delta2 BMI  z-score | 0.89  (0.67, 1.18) | 0.4131 | 0.83  (0.61, 1.13) | 0.2272 |

delta1: difference between birth and hospital discharge; delta2: difference between birth and FU2; HC: head circumference; BMI, body mass index; FU2, 2-year follow-up assessment.

In adjusted analysis, beta values are adjusted for gestational age, sex, multiple births, bronchopulmonary dysplasia, sepsis, necrotizing enterocolitis, retinopathy of prematurity, socio-economic status and major brain lesion.
