# Supplementary material for: Association of growth with neurodevelopment in extremely low gestational age infants: a population-based analysis
Source: Eur J Pediatr. 2022 Jul 22;181(10):3673–81. doi: 10.1007/s00431-022-04567-9 (PMC9508205; doi:10.1007/s00431-022-04567-9)
Supplement: Supplementary file 6 — Supplementary file6 (DOCX 16 KB) [file 431_2022_4567_MOESM6_ESM.docx]

**Table 6: Analyses of small for gestational age (SGA) patients (n=141). Association between somatic growth parameters at birth, at hospital discharge, and at 2-year follow-up and MDI/PDI (BSID-II) at age 2 years.**

|  | unadjusted regression | | adjusted regression | |
| --- | --- | --- | --- | --- |
|  | **MDI** | | | |
|  | β (95% CI) | p-value | β (95% CI) | p-value |
| delta1 weight  z-score | **8.51**  **(3.81, 13.22)** | **0.0004** | **8.45**  **(3.89, 13.02)** | **0.0003** |
| delta1 length  z-score | 2.21  (-2.63, 7.05) | 0.3717 | 3.28  (-1.35, 7.91) | 0.1652 |
| delta1 HC  z-score | 0.03  (-3.98, 4.03) | 0.9899 | 0.29  (-3.52, 4.11) | 0.8808 |
| delta1 BMI  z-score | 0.25  (-3.40, 3.89) | 0.8941 | 0.80  (-2.74, 4.34) | 0.6570 |
| delta2 weight  z-score | 3.91  (0.81, 7.01) | 0.0136 | 4.11  (1.17, 7.06) | 0.0062 |
| delta2 length  z-score | 1.97  (-0.98, 4.91) | 0.1911 | 2.23  (-0.58, 5.05) | 0.1198 |
| delta2 HC  z-score | 2.74  (0.12, 5.36) | 0.0404 | 2.38  (-0.15, 4.90) | 0.0648 |
| delta2 BMI  z-score | 2.47  (-0.33, 5.28) | 0.0838 | 2.89  0.22, 5.55) | 0.0339 |
|  | | | | |
|  | **PDI** | | | |
|  | β (95% CI) | p-value | β (95% CI) | p-value |
| delta1 weight  z-score | 3.06  (-1.65, 7.77) | 0.2033 | 4.27  (-0.43, 8.97) | 0.0748 |
| delta1 length  z-score | 3.82  (-1.04, 8.69) | 0.1233 | 4.23  (-0.55, 9.01) | 0.0831 |
| delta1 HC  z-score | 0.84  (-3.33, 5.01) | 0.6944 | -0.90  (-3.19, 4.99) | 0.6663 |
| delta1 BMI  z-score | -2.39  (-6.06, 1.28) | 0.2015 | -1.10  (-4.70, 2.50) | 0.5504 |
| delta2 weight  z-score | **6.01**  **(2.96, 9.05)** | **0.0001** | **6.15**  **(3.17, 9.13)** | **0.0001** |
|  |  |  |  |  |
| delta2 length  z-score | 3.80  (0.86, 6.73) | 0.0111 | 3.74  (0.87, 6.62) | 0.0106 |
| delta2 HC  z-score | 3.40  (0.79, 6.01) | 0.0107 | 2.83  (0.24, 5.41) | 0.0321 |
| delta2 BMI  z-score | **4.99**  **(2.23, 7.75)** | **0.0004** | **5.18**  **(2.49, 7.87)** | **0.0002** |

delta1: difference between birth and hospital discharge; delta2: difference between birth and FU2; HC: head circumference; BMI: body mass index. FU2, 2-year follow-up assessment.

In adjusted analysis, beta values are adjusted for gestational age, sex, multiple births, bronchopulmonary dysplasia, sepsis, necrotizing enterocolitis, retinopathy of prematurity, socio-economic status and major brain lesion.
